# Short Communication: Integrase Strand Transfer Inhibitors Drug Resistance Mutations in Puerto Rico HIV-Positive Individuals

**DOI:** 10.3390/ijerph18052719

**Published:** 2021-03-08

**Authors:** Pablo López, Grissell Tirado, Andrea Arias, Raphael Sánchez, Elliott R. Rodríguez-López, Vanessa Rivera-Amill

**Affiliations:** Basic Sciences Department and RCMI Center for Research Resources, Ponce Health Sciences University/Ponce Research Institute, Ponce, PR 00716-2348, USA; plopez@psm.edu (P.L.); gtirado@psm.edu (G.T.); aarias@psm.edu (A.A.); rsanchez@psm.edu (R.S.); elrodriguez18@stu.psm.edu (E.R.R.-L.)

**Keywords:** HIV-1, integrase resistance mutations, elvitegravir, raltegravir, dolutegravir, bictegravir

## Abstract

The HIV-1 integrase viral protein is responsible for incorporating the viral DNA into the genomic DNA. The inhibition of viral integration into host cell DNA is part of recent therapeutic procedures. Combination therapy with protease and reverse transcriptase inhibitors has demonstrated good synergistic results in reducing viral replication. The purpose of this study is to assess the occurrence of integrase drug resistance mutations from the period comprising 2013 through 2018 in Puerto Rico (PR). We analyzed 131 nucleotide sequences available in our HIV genotyping database, and we performed drug resistance mutation analyses using the Stanford HIV Drug Resistance Database. Twenty-one sequences (16.03%) harbored major or resistance-associated mutations. We identified the Q148HKR, G140S, Y143R, N155H, S147G, and E138EA major drug resistance mutations and the D232DN, T97TA, E157Q, G163GART accessory mutations. We detected high-level drug resistance to Elvitegravir and Raltegravir (76.19% and 85.71%). Moreover, we identified sequences harboring drug resistance mutations that could provide resistance to Dolutegravir. The transmission of strains with integrase antiretroviral resistance has been previously documented in treatment naïve patients. Given the increase of patients treated with integrase inhibitors, surveillance of drug resistance mutations is an essential aspect of PR’s clinical management of HIV infection.

## 1. Introduction

Approximately 49,791 people have been diagnosed with human immunodeficiency virus type 1 (HIV-1) in Puerto Rico (PR) from 1981 to July 2019 [1]. Puerto Rico is one of the ten states and territories with the highest HIV-1 diagnosis rates, prevalence of infections, and accumulated cases of acquired immunodeficiency syndrome (AIDS) in the United States of America [2]. In the last decade, HIV-1 treatment in PR has primarily consisted of combining protease inhibitors (PIs) and reverse transcriptase inhibitors (RTs). Nevertheless, recent studies have reported a high prevalence of drug resistance mutations (DRMs) to PIs and RTs on the island [3,4,5]. In fact, the presence of DRMs is mainly attributed to low adherence to antiretroviral regimens, which has been shown to increase the chance of therapeutic failure and poor clinical outcomes [6,7]. Therefore, improved management of antiretroviral therapy (ART) is a critical prerequisite for successfully treating HIV-1 positive individuals [8].

The development of new drugs that target different phases of the HIV-1 replication ensures the efficacy of ART regimens [9]. Drugs that target and block viral DNA integration have been included in patients’ antiretroviral regimens [10]. Specifically, these drugs block the mechanism of action of the HIV-1 integrase by binding to its active site, thus preventing the strand transfer activity of the protein and the integration of the viral DNA into the host cell genome, which ultimately abrogates viral replication [10,11,12]. These drugs are known as integrase strand transfer inhibitors (INSTIs) and can be divided into first-generation INSTIs (which include Raltegravir (RAL) and Elvitegravir (EVG)) and second-generation INSTIs (which include Dolutegravir (DTG), Bictegravir (BIC) and Cabotegravir (CAB) [13,14].

The previous standard treatment for HIV-positive individuals involved the use of a combination of at least three ART drugs belonging to five different classes of medications [15]. Current treatment includes a two-drug regimen for HIV-patients who are treatment naïve. The use of HIV-1 integrase inhibitors as first-line antiretroviral therapy should display a relatively high genetic barrier to resistance, maintain low HIV viral loads, confer less central nervous system side effects, be minimally toxic and have low interactions with other drugs [16]. The Food and Drug Administration (FDA) approval of RAL, EVG, and DTG as single pill formulations occurred in 2007, 2014, and 2013, respectively. Moreover, in 2018 a single-tablet regimen of Bictegravir/Emtricitabine/Tenofovir Alafenamide (BIC/FTC/TAF) was also approved by the FDA [17]. Nevertheless, even with multiple highly efficient INSTIs, the emergence of drug resistance mutations has been shown to compromise treatment efficacy [18]. Their prevalence has been reported in both treatment-naïve and treatment-experienced patients, which could become a threat to the success of antiretroviral therapies and demonstrates the importance of consistent genotypic surveillance in clinical management [19]. The combination of integrase strand transfer inhibitors (INSTIs) with the established PIs and RTIs has been demonstrated to provide good synergistic results reducing viral replication [10,20].

In Puerto Rico, most of these INSTIs are included in patients’ antiretroviral regimens as treating physicians are encouraged to follow the HIV Treatment Guidelines for Adults and Adolescents published by the Federal Public Health Services (PHS). In this study, we examined INSTIs-associated drug resistance mutations from samples evaluated in our laboratory from 2013 through 2018 to determine the occurrence of drug resistance mutations to these inhibitors.

## 2. Methods

### 2.1. Ethics Statement

The current study was conducted in accordance with the Declaration of Helsinki, and the protocol was certified by the Institutional Review Board of the Ponce Research Institute to be exempt from the federal policy for the protection of human subjects under the provision of use of existing data and specimens (protocol number 2005039159).

### 2.2. Nucleotide Acid Purification and PCR Amplification

We analyzed the nucleotide sequence data of the HIV-1 integrase gene associated with HIV-1 Puerto Rican isolates (*n* = 131), which were processed with our WHO-accredited HIV-1 genotyping protocol. Briefly, venous blood was obtained using EDTA Vacutainer tubes and centrifuged to collect plasma. The viral RNA was purified using the QIAmp Viral RNA Kit (QIAGEN, USA), following the manufacturer’s instructions. For the first-round RT-PCR amplification, we used the OneStep RT-PCR Kit (QIAGEN, USA) according to the manufacturer’s instructions using primers: 5′-CACAAAGGAATTGGAGGAAATGAAC-3′ (forward) and 5′-CCTAGTGGGATGTGTACTTCTGAAC-3′ (reverse). Thermal cycling conditions for first-round RT-PCR consisted of reverse transcription at 50 °C for 40 min, inactivation at 95 °C for 15 min, followed by 35 cycles of amplification at 94 °C for 30 s, 53 °C for 30 s, and 72 °C for 2 min, with a final extension at 72 °C for 10 min. The first-round PCR amplifies the region corresponding to positions 4164 to 5219 relative to the reference sequence HXB2. A second-round PCR was performed using FastStart PCR Master (Sigma-Aldrich), according to the manufacturer’s instructions utilizing 1 µL of the first-round RT-PCR as a template. Amplification was done using forward primer 5′-ATAAATTAGTCAGTGCTGGAA-3′ and reverse primer 5′-GCTTTCATAGTGATGTCTATA-3, with PCR conditions as follows: a denaturing step at 95 °C for 15 min, followed by amplification for 35 cycles at 94 °C for 30 s, at 48 °C for 30 s, and 72 °C for 2 min, with a final extension at 72 °C for 10 min. The second-round PCR amplified the region corresponding to positions 4195 to 5178 relative to the reference sequence HXB2.

### 2.3. Sequencing Analyses

Sequence data were obtained using an ABI 3730xl automated DNA Analyzer sequencer (Thermo-Fisher Scientific, Waltham, MA, USA). The sequences were 849 bp long (nucleotides 4245–5093 relative to the reference sequence HXB2). Sequences were aligned and edited by using BioEdit software v 7.0.5.2. The HIV-1 subtype was characterized using the REGA subtyping tool v3.0 and confirmed by using COMET HIV-1 [21,22,23]. The genotypic resistance interpretations were performed by using the Stanford HIV Drug Resistance Database program [24]. The Sanger sequences were submitted to Gen Bank with accession numbers: MN002890—MN003021.

## 3. Results

We analyzed one hundred thirty-one HIV-1 integrase sequences corresponding to the period of 2013 through 2018. Demographic data showed that the samples were predominantly from males (60.3%), and the mean ages for males and females were 44 and 49 years, respectively (Table 1). Antiretroviral therapy (ART) experienced patients represented (50.4%) of the sequences whereas (6.1%) were ART-naïve patients, and therapy status was unavailable for (43.5%) of the sequences. According to our data analysis, 99.23% of the sequences were associated with HIV-1 subtype B (*n* = 131); however, one case of HIV-1 subtype A (A1) was identified. This sequence did not harbor any major or accessory drug resistance mutations to INSTIs; however, it presented a polymorphic accessory mutation (L74I) common among 20% of the studied patients but with no significant effect by itself. This finding correlates with previous studies that indicate that about 95–98.9% of the HIV-1 virus in PR is subtype B, with the remainder corresponding to subtypes A (A1, A/E), C, D, F, B/D, and CRF-24BG [25,26]. No transmitted drug resistance (TDR) to INSTIs was detected among the sequences from our ART-naïve patients. Twenty-one integrase gene sequences (16.03%) have major or resistance-associated mutations (Table 2). Similar findings have been observed in the United States during 2009–2012 [27].

**Figure 1:**
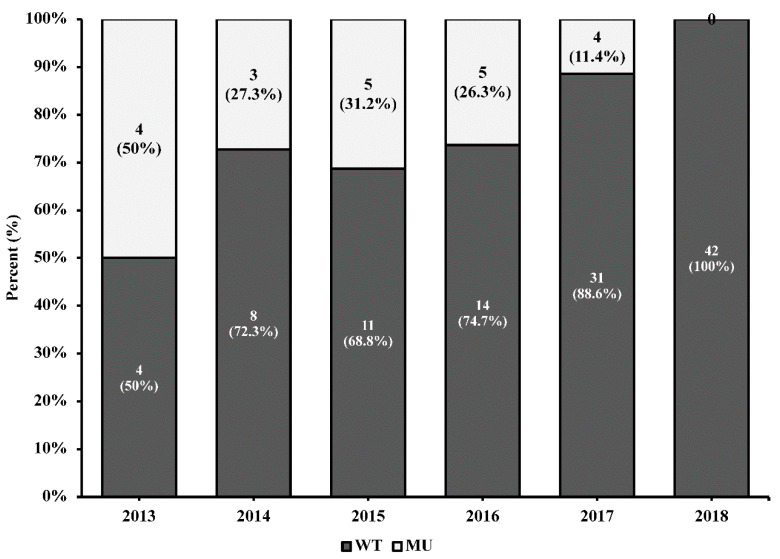
HIV-1 integrase sequences harboring major or accessory drug resistance mutations decrease over time. No INSTIs drug resistance mutations were detected among the 42 sequences analyzed from 2018. The numbers in each bar represent the total number of integrase sequences analyzed.

Intermediate resistance levels then lowered to 25% during 2017 and were not detected in samples from 2018 (Table 2). High-level drug resistance for RAL and EVG was already present in 2013 (75.0%) and reached 80% for EVG in 2015 and 100% for RAL. High-level drug resistance reached 100% for both EVG and RAL by 2016. These levels of high resistance decreased in 2017 to 50% for EVG and 75% for RAL. No high-level drug resistance was detected in 2018. Analysis of the distribution of drug resistance levels by year showed that intermediate drug resistance for BIC and DTG was not detected in 2013 but increased to 33.3% in 2014 and reached 60.0% in samples collected during 2015. Interestingly, we observed a decrease in the frequency of INSTIs major drug resistance mutations compared to the wild-type sequence over the study period (Figure 1).

**Figure 2:**
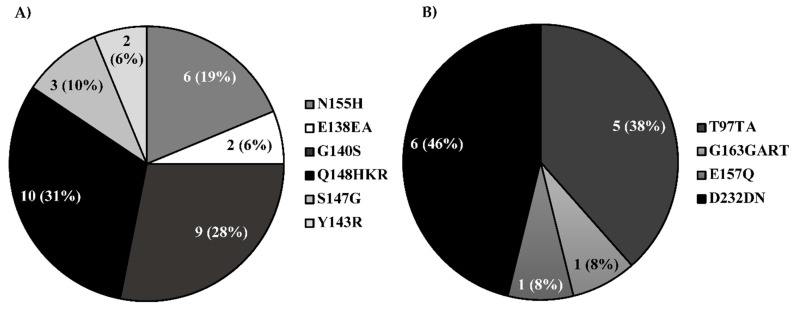
Occurrence of major and accessory INSTI-resistance mutations among HIV-1 patients in Puerto Rico. (**A**) Shows the observed frequency of the integrase mutations Q148HKR, G140S, N155H, S147G, E138EA, and Y143R on our studied samples. (**B**) Frequency of integrase accessory mutations D232DN, T97TA, E157Q, and G163GART detected in patient samples.

The most frequent integrase mutations in our analyzed patient samples were the Q148HKR (31%), G140S (28%), and, although at lower frequencies, we were able to identify the N155H, S147G, Y143R, and E138EA (Figure 2A). The E138EA and G140S mutations by themselves do not reduce or improve INSTIs susceptibility [28,29]. Nevertheless, when combined with Q148QKH, they can provide high-level resistance to RAL or EVG and reduce DTG or BIC efficacy [30,31,32]. Previous studies have demonstrated that HIV-1 patient samples harboring these mutations (E138EA, G140S, and Q148HKR) show high-level resistance to INSTIs [29,33], and these combinations were identified in 1.52% of our sequences. The N155H mutation, associated with high-level resistance to RAL and EVG, was identified in 19% of the samples [34,35]. Meanwhile, the Y143R mutation, found in 6.0% of the sequences, is related to high-level resistance to RAL. The S147G was observed in 10.0% of patients, which moderately reduces susceptibility to this drug [36]. We also identified the resistance-accessory mutations D232DN, T97TA, E157Q, and G163GART (Figure 2B). The D232DN, detected in 46% of the samples with mutations, is an accessory mutation reported in patients receiving EVG and RAL [27,37]. Meanwhile, the T97TA (38%) integrase mutation, when combined with any other major mutation, has been reported to have a synergistic effect, which can reduce susceptibility to these drugs [38].

**Figure 3:**
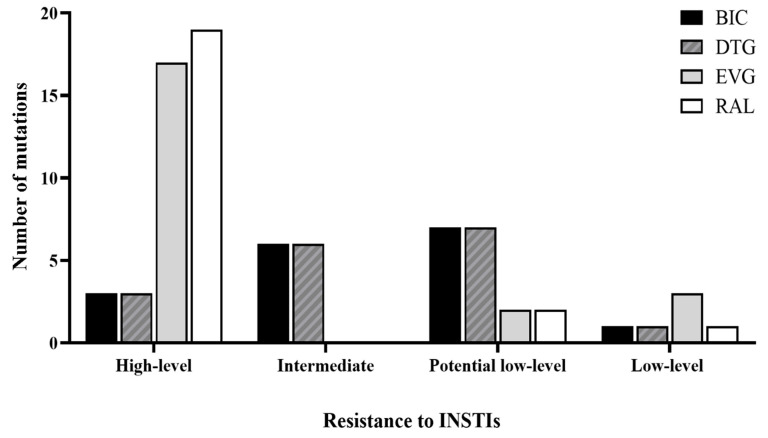
Resistance mutations among HIV-1 patients in Puerto Rico. HIV-1 integrase sequences harboring high-level, intermediate, potential low-level and low-level resistance mutations to Bictegravir (BIC), Dolutegravir (DTG), Elvitegravir (EVG), and Raltegravir (RAL) INSTIs.

Among sequences showing INSTI mutations, we observed drug resistance associated majorly with EVG and RAL (Figure 3). Meanwhile, low-level or potential low-level resistance to DTG and BIC was observed in 38.0% of the samples, whereas 23.8% remained susceptible to the drugs (“wild-type”). Intermediate levels of resistance to BIC and DTG were observed in most samples (Figure 3). Interestingly, two samples, one collected in 2013 and the other in 2016, presented concurrently the mutations E138A, G140S, and Q148H, which confer high-level resistance to all INSTIs, including BIC (Table 2) [39].

## 4. Discussion

The INSTI-resistance mutations percentage is relatively low when compared with mutations that can compromise nucleoside reverse transcriptase inhibitors (NRTIs) and non-nucleoside reverse transcriptase inhibitors (NNRTIs) in Puerto Rican patients during a similar period [25]. This low frequency of drug resistance mutations could be related to the relatively new introduction of these drugs into the ART regimen in PR [40,41]. Several studies have established that a decline in drug resistance mutations possibly reflects improved treatment regimens, which can occur by increasing treatment adherence, care, and support to HIV-1 patients, among other factors [42,43]. However, to confirm this decreasing drug resistance trend on the island, it would be necessary to examine additional sequences from that same period.

Our finding that drug resistance associated mainly with EVG and RAL may be explained by recent studies which established that mutations that provide resistance to RAL could also decrease EVG efficacy [44,45]. Furthermore, RAL and EVG were the first two integrase inhibitors introduced to patients’ antiretroviral regimens in Puerto Rico, which may have allowed more time to develop mutations that could compromise drug susceptibility [46,47]. Low-level resistance to second-generation integrase inhibitors suggests a high genetic barrier for resistance selection. Dolutegravir and Bictegravir are the most recent INSTIs introduced to patients’ regimens on the island. Their advantage is that these drugs show low cross-resistance to mutations that confer resistance to RAL or EVG [48]. However, the presence of three mutations (G140S, Q148H, and S147G) pose a higher risk of failing second-generation drugs [27,49].

While epidemiological and clinical information about these patients was not available for this study, our current analysis provides new information to understand how drug resistance to INSTIs evolves in Puerto Rico. Although the incidence of integrase drug resistance mutations in PR was relatively low and appeared to decrease over the studied period, without close genotypic monitoring, the emergence of drug resistance mutations could increase in the next several years. A recent study established that drug susceptibility and viral fitness may be affected by potential cross-class mutational interactions. Siedner et al. observed that non-nucleoside reverse transcriptase inhibitor resistance before treatment with HIV-1 integrase inhibitors is associated with the long-term failure of integrase-inhibitor-containing first-line regimens [50].

## 5. Conclusions

The use of INSTIs in treatment is an excellent opportunity to improve patient’s clinical outcomes, leading to a healthier lifestyle and reducing the risk of viral transmission [51]. Surveillance of INSTI-resistance mutations is recommended, especially in ART-naïve patients. The constant monitoring of viral evolution and drug resistance mutation dynamics is essential to establish appropriate efforts for controlling the development and expansion of a complex and treatment challenging HIV-1 epidemic in Puerto Rico.

## Figures and Tables

**Table 1:** Demographic characteristics of samples analyzed.

Parameter	
**Sex**	**N (%)**
Male	79 (60.3)
Female	27 (20.6)
Not available	25 (19.1)
**Age**	**Mean (range)**
Male	44 (23–72) ^a^
Female	49 (25–74) ^a^
**Therapy status**	**N (%)**
ART-experienced	66 (50.4)
ART naive	8 (6.1)
Not available	57 (43.5)

^a^ Age data available for sequences of 98% (males) and 93% (females).

**Table 2:** Integrase strand-transfer inhibitor (INSTI) drug resistance mutations identified from 2013 to 2018. Twenty-one sequences (16.03%) obtained from our HIV genotyping database exhibit major drug resistance or resistance-associated mutations. No INSTI mutations were identified in samples from 2018.

		Integrase Mutations		INSTIs Resistance Level			
Year	ID	DRM	Accessory	BIC	DTG	EVG	RAL
2013	235812	N155H	-	PLLR	PLLR	HLR	HLR
	237348	N155H	T97A, D232N	PLLR	PLLR	HLR	HLR
	242798	E138EA, G140S, Q148H	-	HLR	HLR	HLR	HLR
	245847	-	G163GART, D232N	WT	WT	LLR	LLR
2014	255051	-	T97A, D232N	WT	WT	PLLR	PLLR
	255423	G140S, Q148H	-	IR	IR	HLR	HLR
	256143	N155H	-	PLLR	PLLR	HLR	HLR
2015	258076	S147G, Q148H	D232N	LLR	LLR	HLR	HLR
	261110	Y143R	T97TA	WT	WT	LLR	HLR
	262046	G140S, Q148H	-	IR	IR	HLR	HLR
	263288	G140S, Q148H	-	IR	IR	HLR	HLR
	263607	G140S, Q148H	-	IR	IR	HLR	HLR
2016	265677	G140S, Q148H	-	IR	IR	HLR	HLR
	266254	G140S, Q148H, S147G	-	IR	IR	HLR	HLR
	268177	N155H	D232DN	PLLR	PLLR	HLR	HLR
	265852	N155H	-	PLLR	PLLR	HLR	HLR
	266624	E138EA, G140S, Q148H	-	HLR	HLR	HLR	HLR
2017	276082	G140S, Q148H	-	IR	IR	HLR	HLR
	272806	-	T97A	WT	WT	PLLR	PLLR
	273365	S147G, N155H	E157Q, D232N	PLLR	PLLR	HLR	HLR
	273881	Y143R	T97A	WT	WT	LLR	HLR

WT: wild type; PLLR: potential low-level resistance; LLR: low-level resistance; IR: intermediate resistance; HLR: high-level resistance.

## Data Availability

Data generated in this study may be accessed from Gen Bank with accession numbers: MN002890—MN003021.
